# Toxicological Considerations on Local and General Blood-Letting

**Published:** 1829-01-01

**Authors:** 


### TOXICOLOGICAL CONSIDERATIONS AND EXPERIMENTS ON LOCAL AND GENERAL BLOOD-LETTING. By M. VERNIERE, M.D.

The following observations and experiments suggested themselves to the author of this paper (read before the Philomatic Society, on the 16th Aug. 1828) by the fatal accident which befel our country-man, Drake, who died of a poisoned wound, notwithstanding all the resources of the healing art. It is now fully proved, that a tight ligature above the poisoned wound arrests, pro tempore, the entrance of the poison into the general circulation, and, consequently, suspends its deleterious effects. It is the same with the cupping-glass. But during the application of both these, the poison is either kept in situ, or it is becoming blended with the blood of the part, and when the ligature or cupping-glass is removed, it rushes into the torrent of the circulation and destroys life. Hitherto, then, the removal of the poison by excision, or cauterisation, have been the only effectual antidotes. These are cruel and bloody operations—consequently, they are often ineffectually performed. The experiments of Magendie, on Artificial Plethora, as a mean of checking or arresting absorption, are well known. They suggested the following experiment, the details of which we shall greatly abridge.

EXP. 1. Three grains of the alcoholic extract of nux vomica were introduced into a wound made in the paw of a young dog, and a ligature was immediately tightened round the limb, above the humero-cubital articulation. As much warm water was then injected into the jugular vein as the animal could conveniently bear. The ligature was removed, and the animal remained tranquil for half an hour. The ligature was then replaced, and a vein opened below the ligature, whence some blood was received into a glass. The wound was well washed—the ligature removed—and the animal set at liberty, after losing a large quantity of blood from the jugular vein. He continued well. Mean time, the blood which had been taken from the vein below the ligature, was injected (mixed with an equal quantity of tepid water) into the jugular vein of another dog. General tetanus succeeded, and the animal quickly died. Here, it was evident that the blood rising from the poisoned wound was impregnated with the nux vomica sufficiently to destroy the life of the other dog, while the animal into whose veins the warm water was injected, escaped. But injection of warm water into the veins of the human subject, is an operation which would rarely be submitted to by the patient — and which would be reluctantly performed by the practitioner.

EXP. 2. Three grains of the extract were introduced into a wound of the *right* cheek of a dog. The jugular veins were kept moderately compressed for eight minutes, when the *right* jugular was opened, and a large quantity of blood abstracted. The dog was set at liberty, and only suffered from the effects of debility.

EXP. 3. Three grains of the aforesaid extract were introduced into a rather large wound made in the integuments of the abdomen of a dog. The wound did not bleed, although a cupping-glass, with a pump, was kept working over it, for six minutes, when the cupping-glass was removed, and the wound most carefully washed. The animal was quickly seized with convulsions, and died. In this case, the cupping-glass only suspended the action of the poison. The local circulation became impregnated, and when the interruption was removed, the poison acted. The foregoing case pointed to another and important indication—the abstraction of blood from the part acted on by the glass, or below a ligature.

EXP. 4. Three grains of the above-mentioned extract were introduced under the skin of a dog's foot, a tight ligature being made on the same member. After the ligature had been on five minutes, the poison was very carefully washed out of the wound; and this done, the ligature was taken off, and the dog set at liberty. At first he walked about quietly, but soon was seized with convulsions of a tetanic kind, and very severe. A large detraction of blood was made from the jugular vein. In half a minute the convulsions ceased—the dog got up—walked about—and only shewed some wheezing in his breathing, which soon went off.

In the above case, the ligature confined the poison to the vessels of the limb—and, notwithstanding the care which was taken to wash the wound, the quantity imbibed by the vessels of the part, was sufficient, when the ligature was taken off, to produce tetanic convulsions. The other conclusion is of an important therapeutical nature. As far as one case goes, the detraction of a large quantity of blood from the general circulation, carries off so much of the poison—renders the remainder so dilute—or so weakens the impression of it on the nervous system, as to render it innocuous. In cases where venesection fails to afford relief, we have no resource but venesection and transfusion of blood, at the same time. These observations and suggestions apply, of course, only to those poisons which are absorbed rapidly, as the vegetable and animal poisons, and not to those that are of a corrosive quality, and destroy the texture of the parts to which they are applied.—*Repertoire, Oct.* 1828.

